# Supplementary material for: Decay and damage of therapeutic phage OMKO1 by environmental stressors
Source: PLoS One. 2022 Feb 23;17(2):e0263887. doi: 10.1371/journal.pone.0263887 (PMC8865689; doi:10.1371/journal.pone.0263887)
Supplement: S1 File — This supplemental file includes further details on statistical analyses of heat, urea, and saline stress assays reported in the main text. Additionally, it includes visualization and statistical analyses of additional data from heat, urea, and saline stress assays not included in the main text. (DOCX) [file pone.0263887.s001.docx]

# Decay and damage of therapeutic phage OMKO1 by environmental stressors

Michael Blazanin^1,⊗,*^, Wai Tin Lam^1,⊗^, Eli Vasen^1^, Benjamin K. Chan^1^, and Paul E. Turner^1,2^

^1^Department of Ecology and Evolutionary Biology, Yale University, New Haven, CT 06511, USA.

^2^Program in Microbiology, Yale School of Medicine, New Haven, CT 06520, USA.

^⊗^These authors contributed equally to the work, and are listed in alphabetical order.

*Correspondence to: Mike Blazanin, Department of Ecology and Evolutionary Biology, Yale University, 165 Prospect St, New Haven, CT, 06520, USA; mike.blazanin@yale.edu

Keywords: bacteriophage, thermotolerance, virus

Short title: Tolerance of OMKO1 to environmental stress

**Supplemental Material**

**Statistical Tables for Main Text Figures**

**Table S1. Phage OMKO1 suppression of peak bacterial density is robust to presence/absence of heat-shocked media in controls.** Two-sample one-tailed unequal variance t-tests between both positive controls (+ Ctrl is bacteria alone, + Ctrl Shock is bacteria alone in media shocked at 70°C for 360 mins) and each of the 0 minutes heat shock populations show Bonferroni-corrected (10 tests) significant reductions in peak bacterial growth.

| **Contrast** | **Estimated Difference** | **df** | **t-value** | **Bonferroni-adjusted p-value** |
| --- | --- | --- | --- | --- |
| + Ctrl – 0 mins Phage A | -1.24 | 5.35 | -106.6 | <0.001 |
| + Ctrl – 0 mins Phage B | -0.99 | 6.38 | -65.2 | <0.001 |
| + Ctrl – 0 mins Phage C | -1.26 | 5.77 | -105.6 | <0.001 |
| + Ctrl – 0 mins Phage D | -1.27 | 5.24 | -109.2 | <0.001 |
| + Ctrl – 0 mins Phage E | -1.25 | 6.29 | -101.8 | <0.001 |
| + Ctrl Shock – 0 mins Phage A | -1.16 | 5.17 | -68.9 | <0.001 |
| + Ctrl Shock – 0 mins Phage B | -0.90 | 6.98 | -46.6 | <0.001 |
| + Ctrl Shock – 0 mins Phage C | -1.18 | 5.38 | -69.1 | <0.001 |
| + Ctrl Shock – 0 mins Phage D | -1.18 | 5.11 | -70.5 | <0.001 |
| + Ctrl Shock – 0 mins Phage E | -1.17 | 5.68 | -67.7 | <0.001 |

**Table S2. Multiple regression shows saline concentration does not accelerate phage decay rate.** Parameter estimates of the rate of decay over time (slope) depending on saline concentration were evaluated to detect increased decay (decreased slopes) against the null hypothesis of 0 slope using one-tailed t-tests (df = 23) and a Bonferroni correction (5 tests).

| **Saline Concentration (M)** | **Estimated Coefficient** | **t-value** | **Bonferroni-adjusted p-value** |
| --- | --- | --- | --- |
| 0 | 0.013 | 4.41 | 1 |
| LB (0.17) | 0.0029 | 1.90 | 1 |
| 0.5 | 0.0059 | 1.96 | 1 |
| 3 | 0.0029 | 0.95 | 1 |
| 5 | 0.00090 | 0.30 | 1 |

**Table S3. Multiple regression shows nonsignificant acceleration of OMKO1 decay by urea.** Parameter estimates of the rate of decay over time (slope) depending on urea concentration were evaluated to detect increased decay (decreased slopes) against the null hypothesis of 0 slope using one-tailed t-tests (df = 15) and a Bonferroni correction (5 tests). Note that all measures below the limit of detection were excluded from analysis, limiting the power especially at 3M and 4M.

| **Urea Concentration (M)** | **Estimated Coefficient** | **t-value** | **Bonferroni-adjusted p-value** |
| --- | --- | --- | --- |
| 0 | 0.0014 | 0.58 | 1 |
| 1 | -0.0020 | -0.42 | 1 |
| 2 | -0.0020 | -0.42 | 1 |
| 3 | -0.0093 | -1.13 | 0.69 |
| 4 | -0.017 | -2.00 | 0.16 |

**Supplemental Methods & Results**

**
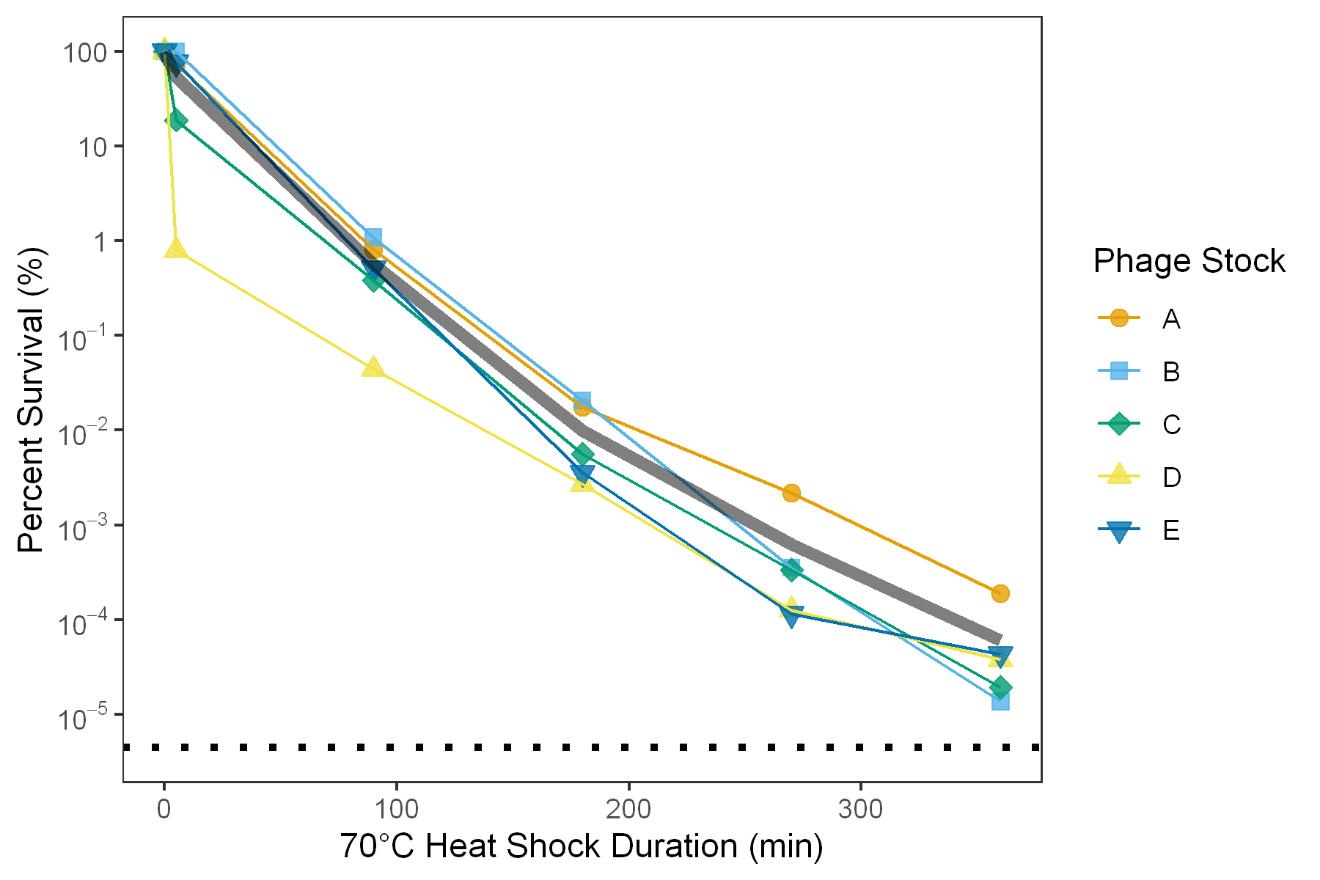
**

**Figure S1. Inactivation of OMKO1 by 70°C thermal stress.** To measure phage particle survival of heat stress over longer periods of time, five biological replicate stocks of phage OMKO1 were exposed to 70°C for 5, 90, 180, 270, or 360 minutes, then titered. Percent survival is plotted relative to the source stock titer. The dotted line denotes the mean limit of detection across all five batches. This plot shows the same data as Fig 1B, now with phage stock information shown. The gray line denotes the overall average percent survival, as plotted in Fig 1B.


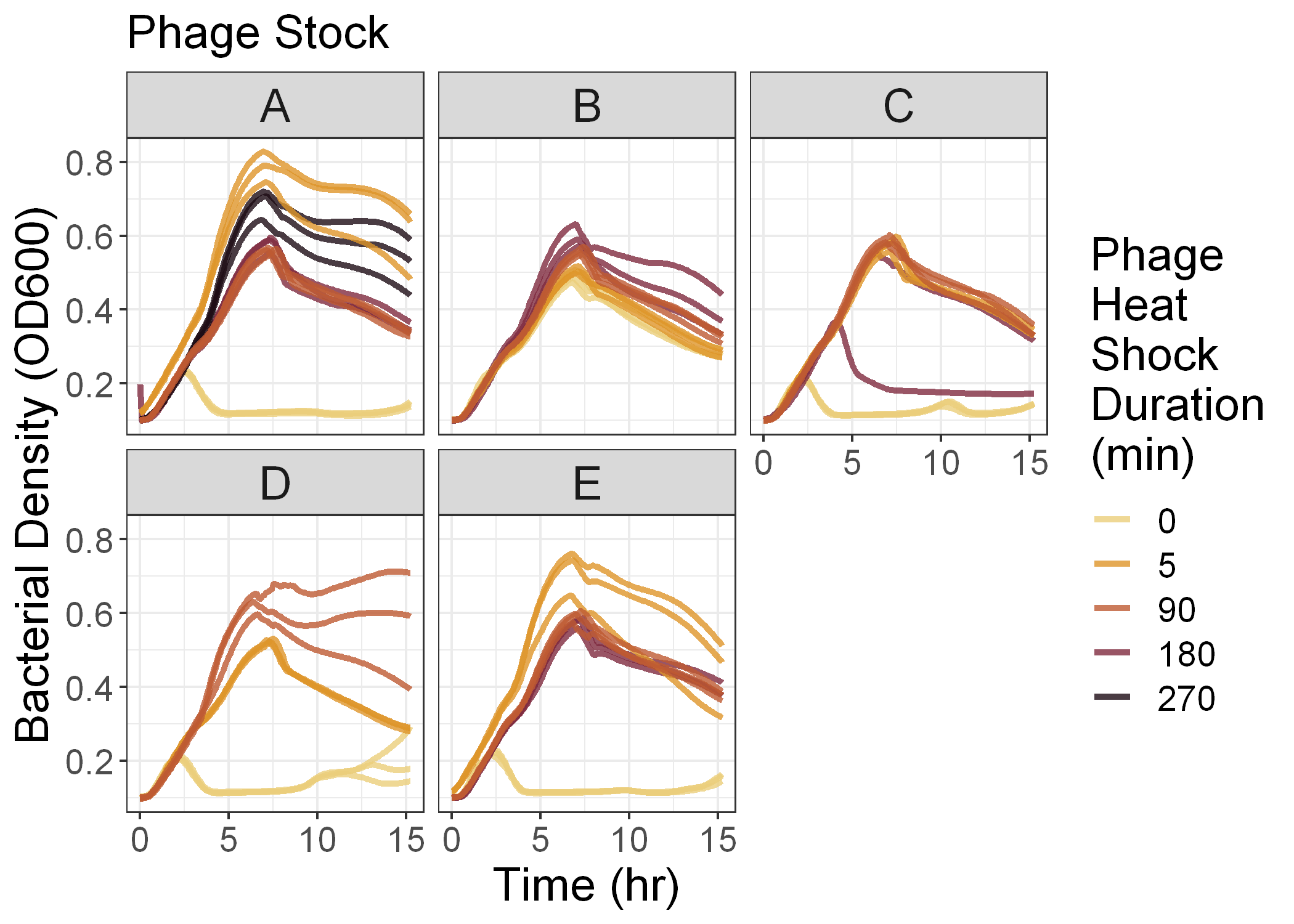


**Figure S2. Bacterial growth curves in presence of post-heat shock normalized phages.** Five different phage OMKO1 stocks were exposed to heat stress at 70°C for variable amounts of time, then normalized to have the same number of plaque-forming-units per volume. Bacteria and phages were inoculated into triplicate wells at constant MOI and the density of bacteria over time is plotted.

In the main text, we report the results of growth curve experiments where post-heat stress phage populations were normalized to a known concentration and grown with bacterial cells (Fig 1C). Because of low survival, at 270 and 360 minutes some stocks could not be normalized, so those timepoints were excluded from analysis and visualization. Here we present all the growth curve data, including combinations which were not inoculated with 200 pfu and 2×10^7^ cfu (Fig S3).


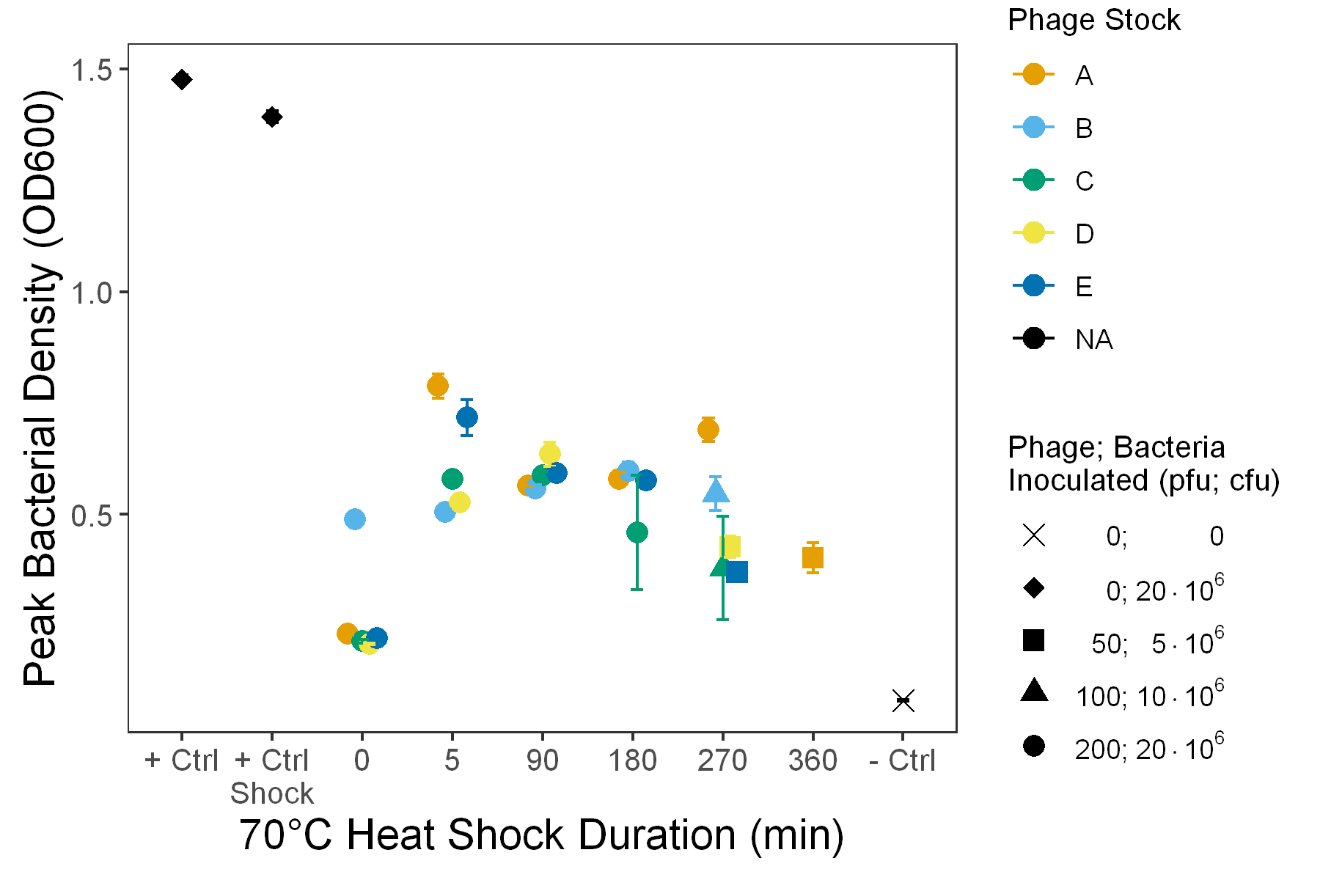


**Figure S3. Fitness suppression of OMKO1 by thermal stress across all tested conditions.** To determine whether phage growth was altered by exposure to heat stress among surviving phage particles, heat shocked phage particles (from Fig 1B) were inoculated with bacteria and grown overnight with repeated measurements of bacterial density. The height of the peak bacterial density was computationally determined and is plotted. Thus, this peak height is a reverse proxy for phage fitness. Error bars denote 95% confidence intervals among replicate wells. Positive control bacteria were grown in the absence of phage in LB media (“+ Ctrl”) or LB media that had been heat shocked for 360 mins at 70°C (“+ Ctrl Shock”), while negative control wells contained only media. Note that, due to low survival after long durations of heat shock, some growth curves were initially inoculated with reduced numbers of bacteria and phage (holding MOI constant), confounding observed differences in peak bacterial density.

In addition to the triplicate measures of the effect of saline and urea stress on phage stability reported in the main text, we also carried out measures of their effects on a single stock with greater sampling over time and a greater range of concentrations.

To measure these effects, 10 μL of phage stock A was added to 1 mL of either urea or salt solution at a defined concentration, and vortexed to mix thoroughly. After a specified time, 50 μL were removed and immediately diluted 200-fold to terminate the stress condition. Then, the stressed samples were titered to calculate survival following stress. Controls (LB with 0.17M saline for saline stress, 0M for urea stress) were titered singly, while non-controls were titered in triplicate. We then fit a generalized linear model and carried out an ANCOVA of log_10_(percent survival) as a function of concentration (as a factor) and the interaction between concentration and time. Values below the limit of detection and treatments that had fewer than 3 points above the limit of detection were excluded from statistical analyses and plots.

For salt stress, we exposed samples of phage OMKO1 to seven different NaCl concentrations ranging from 0M to 5M saline, including LB (0.17M saline) as a control. In each concentration treatment, subsamples were obtained at 5, 30, 60, and 90 minutes. We then measured the number of viable phage particles over time, and compared those densities to the density of phages at 5 minutes in the control to calculate percent survival. Saline concentration did not significantly alter the rate of particle decay over time (slope of the lines in Fig S4; ANCOVA: F(8, 16) = 0.93, p = 0.52).


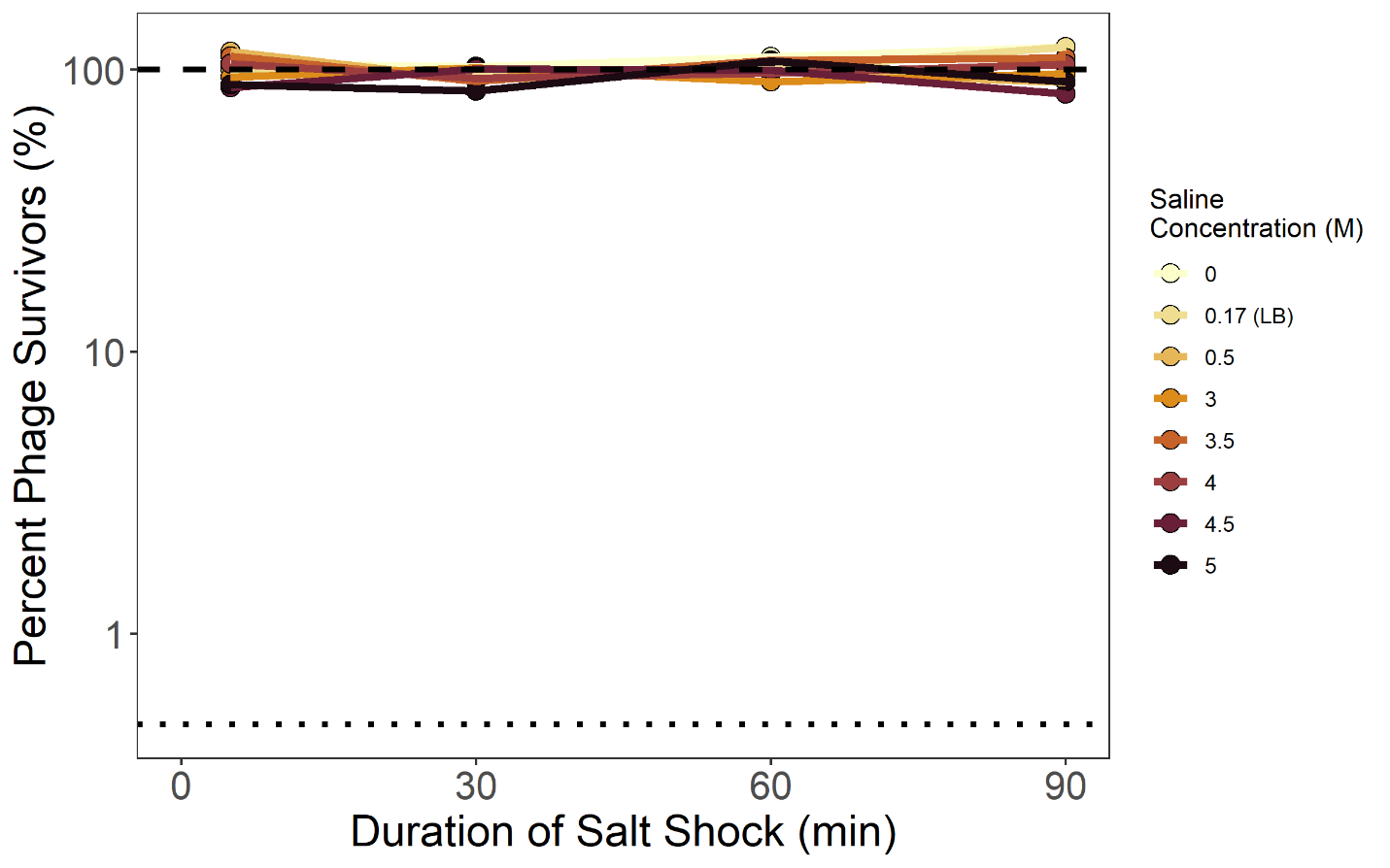


**Figure S4. Phage OMKO1 stability is unaffected by saline concentration**. To measure phage particle survival of saline stress, phage OMKO1 was exposed to a range of salt concentrations then titered over time. Mean percent survival was calculated and is plotted relative to the titer of the LB control (0.17M saline) at 5-minutes. The dashed line denotes 100% survival, while the dotted line denotes the limit of detection.

For urea stress, we exposed samples of phage OMKO1 to eleven different urea concentrations ranging from 0M to 10M, including 0M as a control. In each concentration treatment, subsamples were obtained at 5, 30, 60, and 90 minutes. We then measured the number of viable phage particles over time, and compared those densities to the density of phages at 5 minutes in the control to calculate percent survival. Higher urea concentrations significantly increased the rate of decay of phage particles (slope of the lines in Fig S5; ANCOVA: F(11, 12) = 174.4, p < 0.001). In particular, we observe significantly accelerated decay in all concentrations of Urea greater than 0M (p ≤ 0.01, Table S4), with decay so rapid in concentrations of 6M and above that most measures of phage density in these treatments fell below the limit of detection.

**
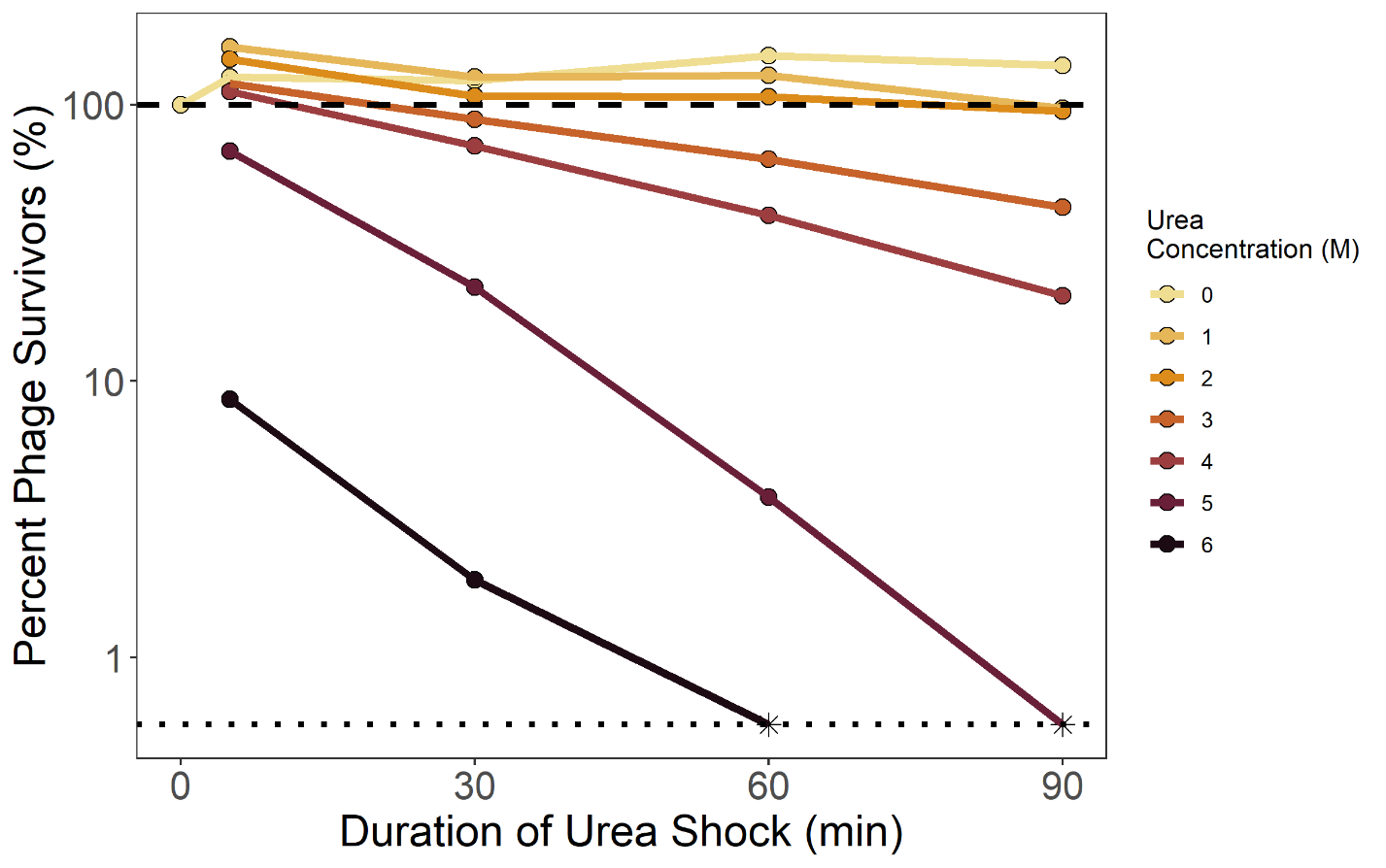
**

**Figure S5. Urea accelerates decay of phage OMKO1.** To measure phage particle survival of urea stress, phage OMKO1 was exposed to a range of urea concentrations then titered over time. Mean percent survival was calculated and is plotted relative to the titer of the control (0M) at 0 minutes. The dashed line denotes 100% survival, the dotted line denotes the limit of detection, and asterisks denote observations that fell below that limit. Concentrations 7M through 10M were tested but caused such rapid particle decay that all measures fell below the limit of detection, and are not plotted.

**Table S4. Multiple regression shows significant acceleration of OMKO1 decay by urea.** Parameter estimates of the rate of decay over time (slope) depending on urea concentration were evaluated to detect increased decay (decreased slopes) against the null hypothesis of 0 slope using one-tailed t-tests (df = 12) and a Bonferroni correction (6 tests). Note that treatments with 6M through 10M urea were excluded from this analysis since they did not have at least three values above the limit of detection

| **Urea Concentration (M)** | **Estimated Coefficient** | **t-value** | **Bonferroni-adjusted p-value** |
| --- | --- | --- | --- |
| 0 | 0.0014 | 2.56 | 1 |
| 1 | -0.0023 | -3.64 | 0.01 |
| 2 | -0.0020 | -3.08 | 0.002 |
| 3 | -0.0052 | -8.26 | < 0.001 |
| 4 | -0.0087 | -13.7 | < 0.001 |
| 5 | -0.23 | -22.0 | < 0.001 |
