# Supplementary material for: Decay and damage of therapeutic phage OMKO1 by environmental stressors
Source: PLoS One. 2022 Feb 23;17(2):e0263887. doi: 10.1371/journal.pone.0263887 (PMC8865689; doi:10.1371/journal.pone.0263887)
Supplement: Attachment — Submitted filename: Response to Reviewers V3.docx [file pone.0263887.s002.docx]

**Response to reviewers**

**We thank the reviewers and editors for their thoughtful feedback on the manuscript. Our revisions have been delayed by the Covid-19 pandemic, but we have now completed revising our manuscript. In particular, we have collected new data, and responded to all reviewer comments included below. For readability, responses are bolded. We believe this greatly strengthens the manuscript and should address all concerns the reviewers expressed about the soundness of our data, analysis, and conclusions.**

1. Is the manuscript technically sound, and do the data support the conclusions?

Reviewer #1: No

Reviewer #2: Partly

Reviewer #3: No

2. Has the statistical analysis been performed appropriately and rigorously?

Reviewer #1: I Don't Know

Reviewer #2: I Don't Know

Reviewer #3: No

3. Have the authors made all data underlying the findings in their manuscript fully available?

Reviewer #1: Yes

Reviewer #2: Yes

Reviewer #3: Yes

4. Is the manuscript presented in an intelligible fashion and written in standard English?

Reviewer #1: Yes

Reviewer #2: Yes

Reviewer #3: No

5. Review Comments to the Author

**Reviewer #1:** The report of Blazanin et al., was aiming to characterize the tolerance ranges of the lytic phage OMKO1 in terms of salinity, urea and temperature. The whole experimental process was unfortunately rather extemporaneous and needs to be entirely revised. The results are very limited and some controls have not been properly selected leading to incomplete conclusions. Additionally, although such information can be useful to know when we are talking about phage therapy candidates, I do not think it is adequate information to stand alone for a publication. They could be though included as complementary data in a phage characterization article.

**We thank the reviewer for their helpful comments on the manuscript, and have addressed and responded to all of them below. Of note, we have collected additional data, and revised our language throughout the methods and results for clarity.**

Line 19: I would not think that ¨seldom studied¨ is a fair term to use here, since heat sensitivity have been a quite commonly studied feature in a big number of novel phage characterization papers.

1. **We agree with the reviewer and have now revised the sentence to read** **“****Just as the effect of environmental conditions (e.g. elevated temperature) on antibiotic efficacy is well-studied, the effect of environmental stressors on the potency of phage therapy candidates demands examination.”**

Line 19: I would recommend using virulent rather than ¨therapeutic¨.

2. **“Virulent” is a description of the phage life history, and would be an accurate description of OMKO1. However, here we aren’t trying to describe the phage life history but it’s medical utilization. Since OMKO1 has been, and continues to be, used therapeutically for human patients, e.g.** [1]**, we feel that “therapeutic” is an appropriate adjective.**

Line 20: The meaning of this sentence is not clear, please rephrase. Is it somehow implied that OMKO1 recovers antibiotic-sensitivity for Pseudomonas? Then you may consider to elaborate a bit more on that.

3. **We thank the reviewer for pointing out this unclear sentence. We’ve now removed this phrase from the abstract of the paper, as it is not essential for that context. In the main text we elaborate in greater detail in the Introduction lines 62 – 69 on this point. Previously published data shows that the administration of phage OMKO1 to *P. aeruginosa* selects for the evolution of phage resistant mutants which also have antibiotic re-sensitivity in *P. aeruginosa*, see** [2,3]**.**

Line 21: Again, I would not consent to the use of word ¨therapeutic¨ as a characterization of a phage. Consider rather virulent or lytic. This rebuttal applies to the entire manuscript.

4. **See response #2**

Line 27: So far it seems that the range of conditions is restricted to salinity and temperature. Yet useful information, cannot stand alone when we the potential of a phage therapy candidate is assessed.

5. **We thank the reviewer for pointing out that the abstract was not sufficiently clear on what stressors we tested. We’ve now added the line: “We assessed the effects of elevated temperatures, saline concentrations, and urea concentrations.” (lines 23-24)

   Additionally, we want to clarify that we view the tests in this manuscript as necessary but not complete tests of a phage’s stability. We explore the effects of saline, urea, and temperature in isolation, but highlight that additional stressors and the interaction of multiple stressors are potential avenues for future work in lines 338-345 of the Discussion**

Line 40: As previously stated, heat sensitivity has been traditionally one of the commonly tested feature for lytic viruses with phage therapy potential. During the last years it is usually omitted though because of the focus switch on molecular analysis and because most application purposes do require expanded temperature margins.

6. **We agree and have revised the sentences to now read** **“With any therapeutic treatment, understanding the effect of environmental conditions on efficacy is paramount. The effects of conditions like high temperature on the stability and application of chemical antibiotics is classically shown [****4-7****].** **In parallel, during development of phage therapy it is important to measure the effects of various environmental conditions on the stability and subsequent reproduction on host cells of candidate therapeutic phages.” (lines 38-42)**

Line 63: citation needed here or further down in line 65 or 67.

7. **Citation to** [2] **(ref #20 in the main text) has been moved from end of previous sentence to end of this sentence (now line 65) to clarify that this paper was the first to report characterization of OMKO1.**

Line 73: citation needed.

8. **The reviewer is correct, and we’ve now added a citation to the clinical trial. The sentence now reads: “****We are also currently testing phage OMKO1 in a clinical trial to resolve or reduce *P. aeruginosa* infections in** **the lungs of CF, non-CF bronchiectasis and COPD patients when administered via aerosol-delivery (nebulizer) treatment (“CYPHY”, NCT04684641).” (lines 73-75)**

Line 120-121: It would be more robust to use biological replicates as well. Also, you need to declare the ¨defined concentration¨ and ¨specified time¨.

9. **We agree with the reviewer and, following this suggestion, have carried out additional data collection now including biological replicates. This data are now shown in Figs 2 and 3. The methods have been revised to include information on concentrations and timepoints, rather than reporting these details in the results. The relevant methods lines now read:
   “****To measure the effects of urea (carbamide) and salt (NaCl) on phage stability, 10 μL of phage stock F, G, or H was added to 990 μL of either urea or salt solution at a defined concentration, and vortexed to mix thoroughly. For saline, these included 0M, 0.5M, 3M, 5M, and an LB control (0.17M). For urea, these included 0M, 1M, 2M, 3M, and 4M. One replicate of each of the three phage stocks was exposed to each concentration. At 45 and 90 minutes, subsamples were obtained by removing 50 μL and immediately diluting 200-fold to terminate the stress condition. Then, the stressed samples were titered in triplicate to calculate survival following stress, with percent survival calculated relative to the control titer (for saline, LB; for urea, 0M) at 0 minutes. We then fit a generalized linear model and carried out an ANCOVA of log_10_(percent survival) as a function of urea or saline concentration (as a factor) and the interaction between concentration and duration. Values below the limit of detection were excluded from statistical analyses.”**

Line 142: If I get this right, you conducted infection experiment using the post-stressed phages. However, I think you should definitely include the normal, wt OMKO1 phages at a similarly low MOI in order to compare the fitness level of the stressed virions. If you did that, it needs to be mentioned as well.

10. **We thank the reviewer for pointing this out, since our phrasing was unclear that these unshocked control phage populations were included in the growth curves experiments. We revised the text to clarify that we ran this control, called the “0 min” shock treatment. We’ve improved our language in lines 206-212 of the Results section to clarify this point, which now reads:
    “****As expected, the presence of unshocked phage OMKO1 (0 min) reduced peak bacterial density relative to the phage-free bacterial controls, indicating that the phage negatively affected bacterial growth (Table S1). Surprisingly, a history of exposure to heat shock reduced the ability of phage particles to suppress bacterial growth, permitting higher peak bacterial densities** **(Tukey Honest Significant Differences among all heat stress treatments showed significant differences between unshocked control of 0 minutes and all other treatments at p < 0.01, see Fig 1C).”**

Lines 161-168: this part is actually material and methods and it was missing from there. Same applies for line 178-184 and lines 206-210.

11. **Content from lines 161-168, 178-184, and 206-210 have now been moved to the methods section. See lines 151-162 (saline), 151-162 (urea), and 119-133 (heat), respectively, in the methods. See lines 240-247 (saline), 256-263 (urea), and 169-192 (heat), respectively, in the results.**

Line 240: As stated above, regular wt virion should definitely be included in the fig 3c experiment and additionally in all presented figures the legends have to be explained.

12. **See response #10 above**

Lines 237-240: this part does not match with the figure content.

13. **Thank you for pointing this out, since our representation of the statistics was not clear. We’ve removed the contrast bars from Fig 1C for clarity and the corresponding text (which this comment referred to) from the figure legend.**

Line 260: I do not really see the rationale behind evaluating the effect of high temperature over a longer time of period since you have already seen that the decrease is quite steep – in a linear way - already during the first 90 min.

14. **We thank the reviewer for pointing out that our rationale for this experiment was insufficiently detailed. We've now clarified our language that our motivation was to observe whether decay would continue exponentially or become biphasic over longer periods of time. The first sentence of the relevant results paragraph, lines 184-186, now reads:
    “****Given the observed substantial decay of phage OMKO1 induced by elevated temperatures, we next sought to observe the dynamics of heat-induced decay over longer periods of time to assess whether decay would continue as exponential or appear biphasic.”

    We also discuss the context of biphasic decay in the discussion section, lines 286-288, which read “****Virus particles decay approximately exponentially (Fig 1B, linear on log-linear axes), with higher temperatures increasing the rate of degradation (Fig 1A), although we have limited resolution to detect a bi-phasic decay, as observed elsewhere [****26****].”**

Line 264: the effect of the stressed virions on their fitness could be of interest to explore. However, such results would not be comparable to something if a control condition i.e. infection at the same MOI with non-stressed virions, is not included.

15. **See response #10 above**

Line 279: Having the issue above risen, the chosen controls are not sufficient to lead to a valid conclusion.

16. **See response #10 above**

Line 280: In an experiment that lasted 270 min, the only ¨significant¨ difference was observed between time point 0 and time point 5 min, whereas from 5min till 270min there was not any statistical difference. Judging by the symbols on the graph I do not really get among which values grounds the statistical analysis was held, but I believe that you can as well realize that there is not any sound conclusion of difference here.

17. **We thank the reviewer for pointing out that our representation and explanation of the statistical differences among the heat shocked and unshocked phages was unclear. Tukey Honest Significant Difference contrasts were carried out between all pairwise combinations of heat shock durations. Thus, 0 minutes (unshocked controls) was significantly different from 5 minutes, 90 minutes, and 180 minutes, while 5 minutes, 90 minutes, and 180 minutes were all not significantly different from each other. We’ve clarified our text in both the main text and the figure legend.

    The figure legend now reads (in part) “****Heat shock treatments that are not significantly different from each other via Tukey Honest Significant Differences are indicated by the same shared letter (a or b).” Lines 237-238

    The main text description of our findings now reads “****Surprisingly, a history of exposure to heat shock reduced the ability of phage particles to suppress bacterial growth, permitting higher peak bacterial densities** **(Tukey Honest Significant Differences among all heat stress treatments showed significant differences between unshocked control of 0 minutes and all other treatments at p < 0.01, see Fig 1C). However, increasing durations of heat shock exposure did not lead to further decreases in phage fitness (Tukey HSD finds no significant differences among 5 minutes through 180 minutes treatments, p ≥ 0.69). Unexpectedly, this remained true even after extremely long heat shocks, where the survival rate was very low (e.g. after 180 minutes survival was ~0.01%; Fig. 1B).” Lines 209-216**

Line 288: Again, we do not know if it is due to decreased virus fitness or just phage resistance due to the extremely low MOI and also due to the absence of actual control.

18. **See response #10 above**

**Reviewer #2:** This study shows the stability of the specific phage of P. aeruginosa OMKO1 under different environmental conditions, such as salinity, urea and heat, and the ability of the phage to grow on the host bacteria after being exposed to an environmental stressor. The results showed in the study suggest that this phage can endure different saline concentrations but is affected by high concentrations of urea and elevated temperatures.

**We thank the reviewer for their helpful comments on the manuscript, and have addressed and responded to all of them below. Of note, we have collected additional data, revised and added to our language in the discussion, and incorporated a new figure in the supplement.**

In my opinion this study is suitable for publication, but only after some considerations:

-Temperature is one of the conditions tested by the authors. The assays use a range of temperatures between 55 and 80 °C; however, none of these temperatures can be expected in the human body. Why are temperatures of 37 °C or even 40°C, which can be expected during fever episodes, not included? In the discussion, the authors say that according to their results temperatures below 50°C will not affect phage OMKO1. In my opinion, is not possible to be sure of that. Especially considering that they use 5 min. at 55°C as their control for 100% survival. The use of a control at 37°C to define 100% survival would be more close to reality.

19. **We thank the reviewer for pointing out that our language was insufficiently clear as to what we meant. We have modified the text to more clearly qualify our conclusions that heat stress specifically should not play a role, based on our data, at temperatures below 55C, since we observe no significant decay over time at 55C. Note that the lack of decay (slope) does not depend on the denominator we use for percent survival, which simply scales the density measures vertically relative to the 5 minute 55C timepoint. The text now reads (lines 346-350):
    “Human body temperatures can reach a maximum of ~40°C, while phage storage conditions could reach perhaps 50°C. We found that phage OMKO1 shows no significant decay over 90 minutes at 55°C (Fig 1A), suggesting that heat stress is only relevant over these timescales at temperatures well beyond those experienced by a therapeutic phage. This, however, does not rule out the possibility of accelerated decay at lower temperatures from factors others than heat stress.”**

-Tolerance to different salinities shows that phages are not affected by these conditions. However, as authors say this experiment was performed only with one biological replicate. Despite that it seems that salinity does not affect the survival of phages, it would be nice if authors can include some biological replicates. The same applies to the experiment of survival to urea. In this case, the authors say that phage samples show “significantly negative rates of decay in the five lowest concentrations (1M through 5M urea)”. I understand that statistical analysis shows that there is a significant rate of decay (slope). However, without biological replicates is not possible to be sure about each point in the data. This is especially important, considering that some of the points are above the control of 100 % of survival, and when biological replicates were used in other experiments, some variation was observed.

20. **We thank the reviewer for this suggestion, and agree that lack of replication weakened the strength of our conclusions. We have now included three biological replicates in both our experiment with salinity and our experiment with urea. These new data confirm our findings that saline has no effect on the stability of OMKO1, while high urea concentrations accelerate the decay of OMKO1. See new data in Figs 2, 3**

-Authors say that biological replicates behave similarly based on the results of the different stocks of phages exposed to heat shock times (figure 3B). However, in the same figure, it’s possible to observe near 100% variation in specific points between the different stocks (first points stock D compared with other stocks of phages). Probably the differences between the stocks (biological replicates) will not alter the tendency of the decay rates in the experiments of heat shock; however, if differences of similar magnitude are present between the biological replicates in the other experiments (for instance, the exposure to urea), this could alter the tendency or rates of decay. The same differences are observed between biological replicates in the experiment to evaluate the phage fitness after heat shock exposure. In my opinion, these differences support the needs of biological replicates in other experiments.

21. **See response #20**

-The authors evaluate the fitness of phages after heat treatment through bacterial growth curves. In this experiment, the PFU concentration of each sample is adjusted in order to add 200 PFU for each well, and using the same MOI in each case. It is expected that the samples exposed to longest heat shock times will have a lower PFU count and more inactivated phages. Therefore I’m wondering if these inactivated phages, which probably are not able to form plaques, maybe they still can block bacterial receptors, or at least part of these phages may be able to block some receptors. This could be contributing to the observed results, and in consequence, the differences in the peak density of the bacterial population will not be entirely explained by the phage fitness, but also by the inactivated phages that are blocking some bacterial receptors. I think the authors should consider this option.

22. **We thank the reviewer for this excellent point, and have incorporated it into our discussion. Given our observation of simultaneous high survival and fitness reduction in two populations at 5 minutes, we conclude that this could explain some, but not all, of the patterns we observe. Lines 292-293 & 297-302 now read:
    “…these results indicate a strong plastic response of phage fitness to high temperature exposure. We propose three possible explanations for this observation…** **The second explanation is that OMKO1 particles which no longer form plaques due to heat stress may inhibit the infection of cells, possibly by competitively binding to phage receptors and blocking infection by “active” phage particles. Although this can explain improved growth of bacteria in the presence of heat-shocked phages, it cannot explain the observed changes when phages have both high survival percentages and low fitness (e.g. populations A and E after 5 minutes, Figs S1, S3).”**

-In the case that phage fitness is affected by heat shock, then I suppose that proteins will be the most affected; therefore, fitness would be affected just during the first round of infection. The new phages released by the first infected bacteria should not have an alteration in their fitness. Then, it would be nice if the authors show the beginning of the infection curve, where differences between the different samples should be visible.

23. **We thank the reviewer for this excellent point. We’ve now included Figure S2, showing the growth curves themselves. As we can see from these data, the beginning of the growth curve does not provide much diagnostic information about the relative fitness of phage strains. Instead, early differences in phage growth and fitness manifest themselves later in the growth curve, in determining the timing and density at which the bacterial population peaks. Such patterns are consistent with yet-unpublished work from our group on bacterial growth curves in the presence of phage, and with previously-published work from our group on bacteria-phage growth curves** [4]**.**

**Figure S2 reproduced below:**

**Figure S2. Bacterial growth curves in presence of post-heat shock normalized phages.** Five different phage OMKO1 stocks were exposed to heat stress at 70°C for variable amounts of time, then normalized to have the same number of plaque-forming-units per volume. Bacteria and phages were inoculated into triplicate wells at constant MOI and the density of bacteria over time is plotted.

Minor comments

Lane 92 “P. aeruginosa strain PA01 was….” The name of the strain should be with an O instead of a zero.

24. **Thank you for pointing this out, we’ve fixed it.**

**Reviewer #3:** Blazanin and collaborators presents a work titled "Characterization the tolerance of phage therapy candidate OMKO1 to environmental stress". Although this paper shows an interesting point (tolerance to abiotic stress) which must be considered in human or other phage therapy applications, on my opinion, the experimental part looks a bit "unrealistic". For example, the authors tested phage stability using temperatures, salt and urea concentrations extremely highs, are they realistic? can you find this concentrations in environmental conditions?- Moreover, the methodology and analysis in a bit confuse, why you take one single replicate during the experiments?

**We thank the reviewer for their helpful comments on the manuscript, and have addressed and responded to all of them below. Of note, we have collected additional data, and revised our language and moved some tables to the supplement for clarity.**

I think the experimental part must be re-done and therefore re-write the manuscript.

25. **Thank you for the suggestion of collecting additional replicates. We agree that it would strengthen our findings, and we have now collected additional replicates for both saline and urea experiments where only a single replicate was collected before. These new data confirm our previous findings: saline concentrations do not accelerate phage OMKO1 decay, urea concentrations do accelerate phage OMKO1 decay.

    We discuss in the discussion section lines 324-337 how the tested environmental ranges related to those that might be experienced during storage or in the human body:

    “Our results are also relevant to the understanding of how environmental stressors might affect use of phage OMKO1 in human therapy. Our experiments were generally designed to exceed the range of environmental conditions that phage particles could experience during storage or within therapy applications in the human body. For example, within the human body the concentrations of salt range from roughly 30mM in urine to 135-145 mM in blood [****42****], all conditions where we saw no increase in phage OMKO1 decay rate (Fig 2); similarly urea concentrations range from 2.6-6.5mM in human blood up to 325mM in human urine [****43****], again conditions where we observed no increase in phage decay rate (Fig 3).** **Human body temperatures can reach a maximum of ~40°C, while phage storage conditions could reach perhaps 50°C. We found that phage OMKO1 shows no significant decay over 90 minutes at 55°C (Fig 1A), suggesting that heat stress is only relevant over these timescales at temperatures well beyond those experienced by a therapeutic phage. This, however, does not rule out the possibility of accelerated decay at lower temperatures from factors others than heat stress. These indications of the general stability of phage OMKO1 despite possible environmental stressors are promising for its further potential uses in clinical applications.”**

Here are my specific comments:

26. **Thank you for these comments, we have fixed these issues**

1.L98. right format for this reference

2. L115-117. Move this sentences to the end of the material and methods sections

3. it is not necessary to inform the definition of moi

4. L153-154. add "()" when you say see R script....

5. L216. I think is very interesting that plaques changes morphology. Please, add a picture showing this.

27. **Unfortunately, we do not have pictures of these plastic plaque morphology changes.**

6. All the tables should be moved to supplementary information

28. **We’ve moved all tables but one to the supplement. We maintain Table 1 in the main text because it is an essential report of our statistical comparisons, and is much more readable than the in-line statistical reporting we would need to add to the main text were it moved to the supplement.**

**Response to Reviewers Citations**

1.	Chan BK, Turner PE, Kim S, Mojibian HR, Elefteriades JA, Narayan D. Phage treatment of an aortic graft infected with Pseudomonas aeruginosa. Evol Med Public Health. 2018;2018: 60–66. doi:10.1093/emph/eoy005

2.	Chan BK, Sistrom M, Wertz JE, Kortright KE, Narayan D, Turner PE. Phage selection restores antibiotic sensitivity in MDR Pseudomonas aeruginosa. Sci Rep. 2016; 1–23. doi:10.1038/srep26717

3.	Gurney J, Pradier L, Griffin JS, Gougat-Barbera C, Chan BK, Turner PE, et al. Phage steering of antibiotic-resistance evolution in the bacterial pathogen, Pseudomonas aeruginosa. Evol Med Public Health. 2020;2020: 148–157. doi:10.1093/emph/eoaa026

4.	Turner PE, Draghi JA, Wilpiszeski R. High-throughput analysis of growth differences among phage strains. J Microbiol Methods. 2012;88: 117–121. doi:10.1016/j.mimet.2011.10.020
