# Supplementary material for: Decay and damage of therapeutic phage OMKO1 by environmental stressors
Source: PLoS One. 2022 Feb 23;17(2):e0263887. doi: 10.1371/journal.pone.0263887 (PMC8865689; doi:10.1371/journal.pone.0263887)
Supplement: Attachment — Submitted filename: Response to Reviewers 2 V2.docx [file pone.0263887.s003.docx]

**We thank the reviewer for their helpful comments on our manuscript. In particular, their responses highlighted an unfortunate misunderstanding about the scope and intent of our work, and we are sorry for any confusion. To address this matter, we have extensively revised the framing of our work in the Introduction and Discussion sections. We believe that these changes, along with the detailed responses included below, address all of the reviewer comments. For convenience, we have included all reviewer comments below, along with our responses in bold. Line numbers refer to lines in the Tracked Changes version, when displayed as “Simple Markup".**

**Response to Reviewer**

1. Reviewer #1: The manuscript “Decay and damage of therapeutic phage OMKO1 by environmental stressors” by Blazanin et al., deals with the analysis of the tolerance ranges of the virulent phage OMKO1 referred to temperature, salinity and urea parameters. Although data included in this study are undoubtedly important, they do not constitute a complete piece of work to guarantee its potential use as therapeutic phage to combat certain infectious diseases due to Gram-negative pathogens.

**We thank the reviewer for stating the importance of this work. We did not intend for our manuscript to comprise “a complete piece of work to guarantee its potential usage as a therapeutic phage”, and we apologize for the unfortunate confusion over the scope of our experiments. We sought to test the tolerance limits of OMKO1 to temperature, salinity, and urea, serving as a general model for understanding the physiological limits of phage particles, with implications for phage therapy since OMKO1 has already been used for this purpose. Indeed, we did not intend for the current study to comprise an explicit or complete test of the tolerance of OMKO1 to therapeutically-relevant stress conditions. To better emphasize this point, we have revised our manuscript (lines 36-43, 76-89, 334-336). We also point to other previously-published papers (citations 20, 21, and 22 from main text) that are more focused on characterization relevant for direct therapeutic use of OMKO1:**

**20.	Chan BK, Sistrom M, Wertz JE, Kortright KE, Narayan D, Turner PE. Phage selection restores antibiotic sensitivity in MDR *Pseudomonas aeruginosa*. Sci Rep. 2016;6:26717. Epub 2016/05/27. doi: 10.1038/srep26717. PubMed PMID: 27225966; PubMed Central PMCID: PMCPMC4880932.**

**21.	Gurney J, Pradier L, Griffin JS, Gougat-Barbera C, Chan BK, Turner PE, et al. Phage steering of antibiotic-resistance evolution in the bacterial pathogen, *Pseudomonas aeruginosa*. Evol Med Public Health. 2020;2020(1):148-57. doi: 10.1093/emph/eoaa026.**

**22.	Chan BK, Turner PE, Kim S, Mojibian HR, Elefteriades JA, Narayan D. Phage treatment of an aortic graft infected with *Pseudomonas aeruginosa*. Evol Med Public Health. 2018;2018(1):60-6. Epub 2018/03/29. doi: 10.1093/emph/eoy005. PubMed PMID: 29588855; PubMed Central PMCID: PMCPMC5842392.**

The above statement is supported by these specific points:

2. Purification and characterization of new phages has been the subject of many articles for several decades. Over time, the focus of these analyses has shifted towards molecular techniques and their possible impact on the virulence of the targeted bacteria. Regarding the phages eventually used in therapy, the characterization of tolerance parameters against stress factors is necessary, but not sufficient, to conclude that a certain phage can be tested in in vivo assays.

**We agree that “characterization of tolerance parameters against [stress] is necessary but not sufficient” for therapeutic phages, and are sorry that this was not evident in the original manuscript submission. We have edited our manuscript (lines 87-89, 334-336) to emphasize this point, including explicit acknowledgement that our experiments should be considered necessary but not sufficient, if characterization of stress tolerances for a therapeutic phage were the goal.**

3. The current manuscript contains valuable information on the relevant tolerance of OMKO1 to a wide range of salt concentrations whereas is affected by high concentrations of urea and elevated temperatures. These latter conditions, however, are not totally significative since some of the urea concentrations and temperatures over 55ºC are simply nonsense, in terms of environmental conditions.

**Please see above response to concern #1. In particular, we intended for this work to serve as a model for understanding the physiological limits of phage particles, with implications for the therapeutic applications of phages, rather than an explicit test of the tolerance of OMKO1 to therapeutically-relevant stress conditions. Thus, our experiments assessed the limits of phage tolerance to severe short-term stressors, which sometimes occurred within the ranges relevant for therapeutic applications, and sometimes occurred beyond such ranges. Since preliminary experiments showed no effects of low urea concentrations and low temperatures on phage survival, we increased the severity of those conditions in order to identify when OMKO1 decay becomes significant. In order to make these points clearer, we have modified our manuscript on lines 21-23, 36-43, 76-89, 272-278.**

4. One surprising issue in the manuscript is the comment about the preparation of “high-titer stocks (lysates)” of OMKO1.
   - Does it mean that the phage is not further purified, normally through a CsCl gradient step? This protocol, or something similar, is practically mandatory to obtain highly purified phage with the highest possible titer. In this sense, what does it mean “high-titer” expressed as PFU/mL?

**The reviewer is correct that OMKO1 stocks were generated through our standard phage culture protocol and not further purified through a CsCl gradient. For the purposes of assessing the stress tolerance limits of phage particles within the lab setting, additional CsCl-gradient purification of phage stocks was deemed unnecessary. Phage stocks were typically above 10e10 pfu/mL. For emergency therapies using OMKO1, including U.S. FDA-approved therapeutic administration, additional purification methods were used to generate highly purified phage stocks. See:**

**22.	Chan BK, Turner PE, Kim S, Mojibian HR, Elefteriades JA, Narayan D. Phage treatment of an aortic graft infected with Pseudomonas aeruginosa. Evol Med Public Health. 2018;2018(1):60-6. Epub 2018/03/29. doi: 10.1093/emph/eoy005. PubMed PMID: 29588855; PubMed Central PMCID: PMCPMC5842392.**

- In addition, how long are the phage lysates stored at 4ºC?

**The titer of the phage stock was measured at the beginning of each assay, to normalize for any changes in phage stock concentration that occurred over time during storage at 4C. Phage stock titers were typically above 10e10 pfu/mL at the beginning of each assay.**

- Is this storage in LB medium, as stated in line 123, without any preservative compound?

**Yes. Our standard laboratory protocol is to store working stocks of the phage at 4C in the growth medium used to culture their bacterial host, in this case LB medium.**

- Have the authors checked the evolution of phage titer over time at 4ºC, -20ºC, and -80ºC with glycerol?

**We agree with the reviewer that understanding the effects of storage on phage titers over time is important. Indeed, changes in phage titers over time under various storage temperatures have been measured, but are beyond the scope of the current study and will be published elsewhere. We do not report any such changes here because the titers of phage stocks stored at 4C were re-measured immediately before each assay, to normalize for any changes over time during storage.**

- Does this phage retain its titer with lyophilization and further resuspension?

**We agree with the reviewer that understanding the effects of lyophilization and resuspension on phage stocks is important. Indeed, the effects of lyophilization and resuspension on OMKO1 are currently being tested, but are beyond the scope of the present study and will be published elsewhere.**

- These details are important for the study of any new phage and are lacking in the manuscript.

**We agree with the reviewer that understanding the effects of storage conditions on a new phage is extremely important. However, the present manuscript was not intended to explicitly test the effect of therapeutically-relevant factors on OMKO1, and so these other effects are beyond the scope of the current study, and are avenues for ongoing and future work to be reported elsewhere.**

5. Once set up the optimal parameters to retain the highest titer, after checked the storage conditions and the tolerance to different stressors, some in vitro experiments are necessary to carry out, particularly when the phage is thought to use it as “therapeutic” to kill susceptible bacterial pathogens. In this case, the targeted bacterium is apparently restricted to Pseudomonas aeruginosa, an important Gram-negative pathogen that causes varied infections, including those affecting lung in cystic fibrosis patients. These latter infections are well known that are produced by P. aeruginosa grown as biofilms. For these reasons, some crucial experiments would be to check whether OMKO1 is capable of reducing the P. aeruginosa viable cells after adding the phage, both in planktonic and biofilm cultures.

**We agree on the importance of such experiments. Fig 1C shows the ability of OMKO1 to reduce *P. aeruginosa* in planktonic cultures, while we report on the ability of OMKO1 to reduce *P. aeruginosa* in both planktonic and biofilm cultures in previously published work:**

**22.	Chan BK, Turner PE, Kim S, Mojibian HR, Elefteriades JA, Narayan D. Phage treatment of an aortic graft infected with Pseudomonas aeruginosa. Evol Med Public Health. 2018;2018(1):60-6. Epub 2018/03/29. doi: 10.1093/emph/eoy005. PubMed PMID: 29588855; PubMed Central PMCID: PMCPMC5842392.**

**We do not include such data on biofilm-killing in this manuscript as it is outside the scope of the present study, which focused on the responses of OMKO1 virus particles to environmental stressors.**

6. Given the rationale of the successive and necessary in vitro assays before reaching the validation in in vivo experiments, or clinical trials on patients, it is rather surprising the comments included in the Introduction section of the manuscript (lines 73-77):

   We are also currently testing phage OMKO1 in a clinical trial to resolve or reduce P. aeruginosa infections in the lungs of CF, non-CF bronchiectasis and COPD patients when administered via aerosol-delivery (nebulizer) treatment.

   While phage OMKO1 already has been used successfully for patient treatment, its stability across environmental conditions remains uncharacterized.

   If I understand correctly, phage OMKO1 has been used in this kind of assays or clinical trials without a complete characterization of stability or other in vitro experiments.

**We agree that much in vitro work is necessary before a phage can be used therapeutically. OMKO1 has undergone extensive in vitro characterization, some of which is reported in:**

**20.	Chan BK, Sistrom M, Wertz JE, Kortright KE, Narayan D, Turner PE. Phage selection restores antibiotic sensitivity in MDR Pseudomonas aeruginosa. Sci Rep. 2016;6:26717. Epub 2016/05/27. doi: 10.1038/srep26717. PubMed PMID: 27225966; PubMed Central PMCID: PMCPMC4880932.**

**21.	Gurney J, Pradier L, Griffin JS, Gougat-Barbera C, Chan BK, Turner PE, et al. Phage steering of antibiotic-resistance evolution in the bacterial pathogen, Pseudomonas aeruginosa. Evolution, Medicine, and Public Health. 2020;2020(1):148-57. doi: 10.1093/emph/eoaa026.**

**22.	Chan BK, Turner PE, Kim S, Mojibian HR, Elefteriades JA, Narayan D. Phage treatment of an aortic graft infected with Pseudomonas aeruginosa. Evol Med Public Health. 2018;2018(1):60-6. Epub 2018/03/29. doi: 10.1093/emph/eoy005. PubMed PMID: 29588855; PubMed Central PMCID: PMCPMC5842392.**

**Other characterization of OMKO1 is ongoing and yet-to-be published. However, the reviewer is correct in understanding that OMKO1 has received U.S. FDA approval for, and usage in, both emergency therapeutic treatments and an ongoing clinical trial (see ClinicalTrials.gov:** [**https://clinicaltrials.gov/ct2/show/NCT04684641**](https://clinicaltrials.gov/ct2/show/NCT04684641)**).**

**Response to Editor**

1. Please ensure that your manuscript meets PLOS ONE's style requirements, including those for file naming. The PLOS ONE style templates can be found at https://journals.plos.org/plosone/s/file?id=wjVg/PLOSOne_formatting_sample_main_body.pdf and

<https://journals.plos.org/plosone/s/file?id=ba62/PLOSOne_formatting_sample_title_authors_affiliations.pdf>.

**We have updated our manuscript and believe it now meets all style requirements**

2.We note that the grant information you provided in the ‘Funding Information’ and ‘Financial Disclosure’ sections do not match.

When you resubmit, please ensure that you provide the correct grant numbers for the awards you received for your study in the ‘Funding Information’ section.

**We have provided our Funding Statement in the cover letter and believe it matches the funding information provided in Editorial Manager.**

3. Thank you for stating the following in the Acknowledgments Section of your manuscript:

“support for Wai Tin Lam from the Chinese University of Hong Kong.”

We note that you have provided funding information that is not currently declared in your Funding Statement. However, funding information should not appear in the Acknowledgments section or other areas of your manuscript. We will only publish funding information present in the Funding Statement section of the online submission form.

Please remove any funding-related text from the manuscript and let us know how you would like to update your Funding Statement. Currently, your Funding Statement reads as follows:

“We disclose financial support for Wai Tin Lam from the Chinese University of Hong Kong.”

Please include your amended statements within your cover letter; we will change the online submission form on your behalf.

**We have removed any mention of funding/support from our Acknowledgements, and provided an updated Acknowledgements in our re-submitted cover letter.**

4. Thank you for stating the following in the Competing Interests section:

“Paul E. Turner discloses a financial interest in Felix Biotechnology, Inc., a phage therapeutic company with first rights to use patents resulting from this work. Paul Turner sits on the Board of Directors of Nextbiotics.”

Please confirm that this does not alter your adherence to all PLOS ONE policies on sharing data and materials, by including the following statement: ""This does not alter our adherence to PLOS ONE policies on sharing data and materials.” (as detailed online in our guide for authors http://journals.plos.org/plosone/s/competing-interests). If there are restrictions on sharing of data and/or materials, please state these. Please note that we cannot proceed with consideration of your article until this information has been declared.

Please include your updated Competing Interests statement in your cover letter; we will change the online submission form on your behalf.

**We have included an updated Competing Interests in our cover letter, adding the statement “This does not alter our adherence to PLOS ONE policies on sharing data and materials.”**

5. We note that you have stated that you will provide repository information for your data at acceptance. Should your manuscript be accepted for publication, we will hold it until you provide the relevant accession numbers or DOIs necessary to access your data. If you wish to make changes to your Data Availability statement, please describe these changes in your cover letter and we will update your Data Availability statement to reflect the information you provide.

**We will provide repository information upon acceptance and note the manuscript will be held upon acceptance until you have received such accession numbers/DOIs**

6. We note that you have included the phrase “data not shown” in your manuscript. Unfortunately, this does not meet our data sharing requirements. PLOS does not permit references to inaccessible data. We require that authors provide all relevant data within the paper, Supporting Information files, or in an acceptable, public repository. Please add a citation to support this phrase or upload the data that corresponds with these findings to a stable repository (such as Figshare or Dryad) and provide and URLs, DOIs, or accession numbers that may be used to access these data. Or, if the data are not a core part of the research being presented in your study, we ask that you remove the phrase that refers to these data.

**We have revised our manuscript to not include “data not shown”.**
